# A short-term increase in cancer risk associated with daytime napping is likely to reflect pre-clinical disease: prospective cohort study

**DOI:** 10.1038/bjc.2012.291

**Published:** 2012-07-10

**Authors:** B J Cairns, R C Travis, X-S Wang, G K Reeves, J Green, V Beral

**Affiliations:** 1 Cancer Epidemiology Unit, University of Oxford, Richard Doll Building, Roosevelt Drive, Oxford OX3 7LF, UK

**Keywords:** daytime napping, sleep disturbance, breast cancer, reverse causation, pre-clinical cancer

## Abstract

**Background:**

Sleep disturbance, a correlate of which is daytime napping, has been hypothesised to be associated with risk of breast and other cancers.

**Methods:**

We estimated relative risks (RR) of breast and other invasive cancers by the reported frequency of daytime napping in a large prospective cohort of middle-aged women in the UK.

**Results:**

During an average of 7.4 years of follow-up, 20 058 breast cancers and 31 856 other cancers were diagnosed. Over the first 4 years of follow-up, daytime napping (sometimes/usually *vs* rarely/never) was associated with slightly increased risks of breast cancer (*RR*=1.10, 95% CI 1.06–1.15) and of other cancers (*RR*=1.12, 1.08–1.15), but the RRs decreased significantly with increasing follow-up time (*P*=0.001 and *P*=0.01, respectively, for trend). Four or more years after baseline, there was no elevated risk of breast cancer (*RR*=1.00, 0.96–1.05), and only marginally greater risk of other cancers (*RR*=1.04, 1.01–1.07).

**Conclusion:**

The effect of pre-clinical disease is a likely explanation for the short-term increased risk of breast and other cancers associated with daytime napping.

Concern about possible increased risks for cancer associated with night work, circadian rhythm disruption, and impaired nocturnal melatonin production (Cohen *et al*, 1978; Stevens, 1987; Erren, 2002; Stevens, 2002; Straif *et al*, 2007; Wang *et al*, 2011) has prompted interest in cancer risk in relation to sleep behaviours and disturbance. Several studies have examined associations between sleep duration and breast cancer risk (Verkasalo *et al*, 2005; McElroy *et al*, 2006; Pinheiro *et al*, 2006; Kakizaki *et al*, 2008; Wu *et al*, 2008), but have not demonstrated a consistent association. Daytime fatigue and daytime napping are associated with sleep disturbance (Ursin *et al*, 2005), and cancer risk might therefore be related to daytime napping. Although there is a substantial literature on sleep problems and fatigue in cancer patients (Davidson *et al*, 2002; Stasi *et al*, 2003; Lee *et al*, 2004), including before treatment (Ancoli-Israel *et al*, 2006), little has been published on whether daytime napping is a marker of pre-clinical disease. The association between daytime napping and cancer mortality has been examined in two cohorts (Suzuki, 2007; Stone *et al*, 2009; Tanabe *et al*, 2010); one found increased liver cancer mortality among women who nap during the day (Suzuki, 2007). This is the first prospective study to investigate the association between daytime napping and cancer incidence.

## Materials and methods

The Million Women Study, a population-based prospective cohort study, has been described in detail elsewhere (The Million Women Study Collaborative Group, 2003). Briefly, during 1996–2001 ∼1.3 million women were recruited through the UK National Breast Screening Programme for a study of women’s health, initially investigating breast cancer risk in relation to use of hormone therapies for the menopause. Ethical approval for the study was provided by the Oxford and Anglia Multi-Centre Research Ethics Committee.

Baseline for these analyses was ∼3 years after recruitment, when participants were invited to complete a second study questionnaire (1999–2005; see http://www.millionwomenstudy.org) with questions on a range of health and lifestyle factors, including: ‘Do you have a nap during the day?’, with possible responses ‘rarely/never’, ‘sometimes’, and ‘usually’. Data on incident cancers during the follow-up period, coded according to the International Classification of Diseases, 10th Revision (ICD-10; World Health Organisation, 1992), are routinely provided for all participants by the National Health Service Central Registers. For this analysis we examined risks of all malignancies combined (ICD-10 C00–C97) and risks of cancers at 17 of the most common sites, particularly breast cancer (ICD-10 C50). Women were excluded from all analyses if they had a previous cancer or missing data on daytime napping at baseline. For analyses of endometrial cancer, women were also excluded if they reported a hysterectomy before baseline, or had unknown hysterectomy status; for analyses of ovarian cancer, women were also excluded if they reported a bilateral oophorectomy before baseline, or had unknown oophorectomy status.

Repeat assessments of daytime napping behaviours were available for some participants, and were used to assess repeatability of reported napping behaviours over time. A small proportion of participants completed the baseline study questionnaire twice, with 1.7 years on average between first and repeat responses. The same question was also asked of all participants 4.5 years later, on average (most during 2006–2007), with possible responses ‘rarely/never’, ‘sometimes’, ‘usually’, and ‘most of the time’ the latter two responses were combined to assess agreement with baseline data. Baseline and repeat responses were compared using Cohen’s kappa statistic for agreement (Cohen, 1960) and Spearman’s rank correlation coefficient.

Relative risks (RR) were estimated by categories of daytime napping at baseline using Cox regression, with minimal adjustment by stratification for age at and region of recruitment, and further adjustment for health and lifestyle covariates, as described below. Analyses were conducted either over the full period of follow-up for incident cancer, or, to investigate napping as a possible marker of pre-clinical disease, after dividing follow-up time from baseline into approximately equal intervals (2-year periods, 0–1.9, 2–3.9, 4–5.9, and ⩾6 years, or 4-year periods, 0–3.9 and ⩾4 years). Tests for trends in RRs with increasing follow-up time were conducted by inverse-variance weighted least squares regression of the log RRs against the average time at risk in each 2-year follow-up period. All statistical tests were two-sided. All analyses were performed using the Stata version 12.0 (StataCorp, College Station, TX, USA).

## Results

In total, 795 238 women aged 50–64 years at recruitment were included in these analyses, with 7.4 years of follow-up per women on average (Supplementary Table 1). During this period, 51 914 incident invasive cancers were diagnosed, including 20 058 breast cancers. Women who reported that they napped either sometimes or usually were older and (after adjustment for age) slept longer on average over a 24-h period than women who reported napping rarely/never; they were more likely to be of lower socioeconomic status, to smoke, and to use menopausal hormone therapy, had a higher body mass index and lower alcohol consumption, and were less likely to engage in strenuous physical activity or be in paid employment. Almost all women were postmenopausal at baseline.

After exploring effects of potential confounders, we adopted a multivariable model for subsequent analyses of RRs in relation to daytime napping at baseline, adjusting for age at recruitment, region of residence, smoking status, body mass index, sleep duration, use of menopausal hormone therapy, socioeconomic status, strenuous physical activity, and alcohol consumption (see Supplementary Table 2 for results and covariate definitions). Repeat self-reports of napping behaviour from 15 235 women, provided 1.7 years on average after baseline, showed good agreement with baseline data (percentage agreement=79%, Cohen’s kappa=0.61; Spearman’s correlation=0.69). There was similar, moderately good agreement of daytime napping reported at baseline with that reported 4.5 years later, on average, among 531 633 women (percentage agreement=74% Cohen’s kappa=0.51; Spearman’s correlation=0.61).

Overall, daytime napping at baseline was associated with a small increase in total cancer risk during follow-up. Under the multivariable model described above, the adjusted RRs of any cancer were 1.06 (95% CI 1.04–1.08) for napping sometimes and 1.11 (1.07–1.15) for napping usually *vs* rarely/never (Supplementary Figure 1).

**Figure 1:**
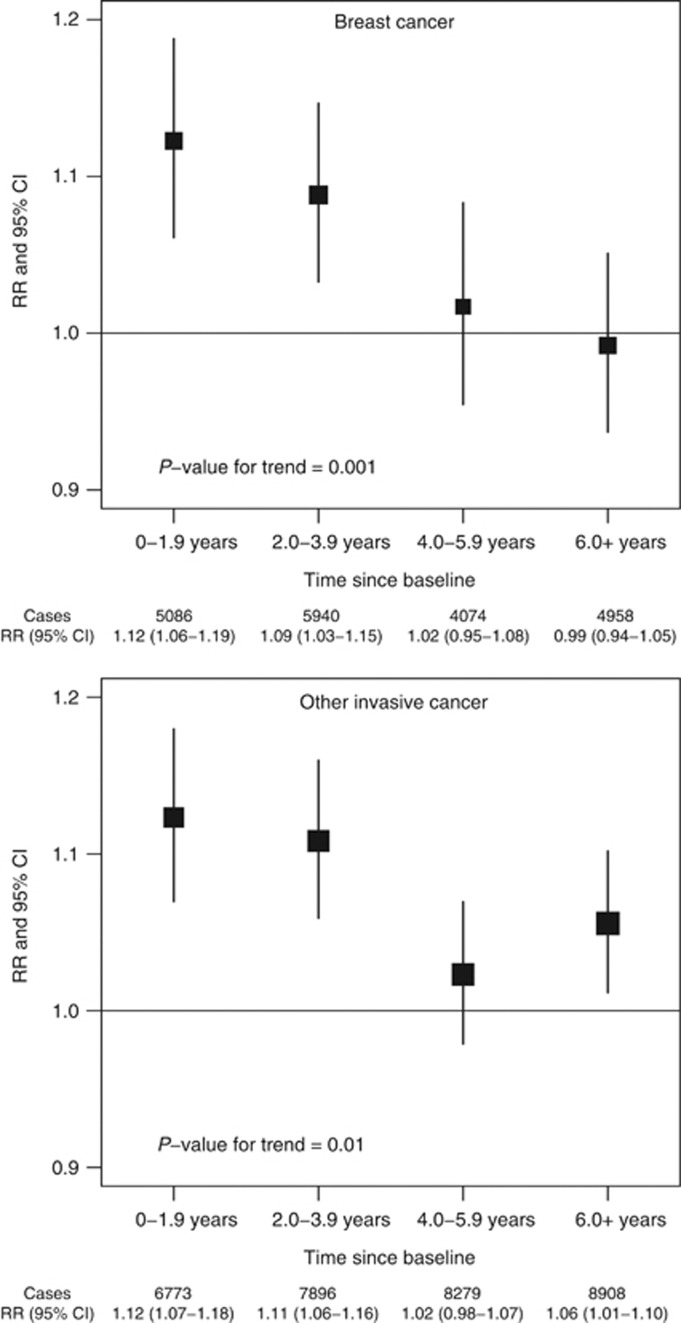
RRs of breast cancer and any other invasive cancer for women who nap sometimes or usually *vs* those who nap rarely/never, by period of follow-up.

**Figure 2:**
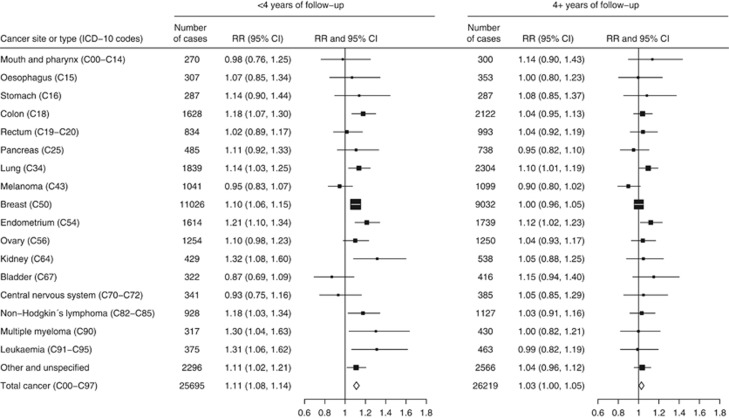
RRs of cancers at 17 specific sites, other cancers and all cancers combined, for women who nap at least sometimes *vs* those who nap rarely/never, by period of follow-up.

We also estimated RRs for the dichotomous comparison of women who reported napping either sometimes or usually *vs* those who reported napping rarely or never, in each 2-year period of follow-up after baseline (Figure 1). Results for breast cancer were considered separately from cancers at other sites. Relative risks declined significantly during follow-up both for breast cancer (*P*=0.001 for trend) and for other cancers (*P*=0.01 for trend). The RR of breast cancer was highest in the first 4 years of follow-up (combined *RR*=1.10, 95% CI 1.06–1.15, for napping sometimes or usually *vs* rarely/never, in the first 4 years after baseline), but after 4 or more years of follow-up (average 7.6 years), there was no significant excess risk associated with more frequent napping (combined *RR*=1.00, 0.96–1.05). A similar pattern was observed in the RRs for invasive cancers other than breast cancer (*RR*=1.12, 1.08–1.15, in the first 4 years after baseline, and *RR*=1.04, 1.01–1.07, 4 or more years after baseline). In detailed investigations by cancer site, associations during the first 4 years after baseline were attenuated 4 or more years after baseline, despite similar case numbers in each period, and became nonsignificant for all sites except the lung and endometrium (Figure 2). Because an association with liver cancer mortality was previously reported (Suzuki, 2007), we also investigated the association of napping with liver cancer incidence. Relative risks were 1.19 (0.84–1.69) and 1.09 (0.82–1.46), respectively, in the first 4 years after baseline and 4 or more years after baseline, not replicating the previous finding.

## Discussion

Pre-clinical disease is a likely explanation for the observed decline over time in the risk of cancer associated with daytime napping. Because good repeatability of self-reported napping behaviour was maintained over time, regression dilution due to reporting errors or changes in napping behaviours would not be expected to produce the observed attenuations of RRs during follow-up. It is unknown whether associations of cancer risk with other sleep or fatigue-related behaviours might also reflect pre-clinical disease.

The main strengths of this study are its large size and prospective design, the virtually complete follow-up for incident cancers, and the ability to examine the repeatability of reported daytime napping several years later. For cancers other than breast cancer, however, follow-up may not be long enough to rule out a residual effect of pre-clinical disease, because some cancers present with specific symptoms later than others. It is also impossible to rule out residual confounding in reported RRs, and in particular we were unable to investigate potential confounding factors related to sleep or napping behaviours, such as chronotype and employment in shift work.

In summary, there was an association between more frequent daytime napping and a slight increased risk of breast and some other cancers in the first 4 years of follow-up in this cohort. This is consistent with napping being a marker of pre-clinical disease in some women.
