# Supplementary material for: A short-term increase in cancer risk associated with daytime napping is likely to reflect pre-clinical disease: prospective cohort study
Source: Br J Cancer. 2012 Jul 10;107(3):527–30. doi: 10.1038/bjc.2012.291 (PMC3405227; doi:10.1038/bjc.2012.291)
Supplement: Supplementary Information [file bjc2012291x1.pdf]

A short-term increase in cancer risk associated with daytime napping is likely to reflect pre-clinical disease: prospective cohort study

ONLINE SUPPLEMENT

Supplementary Tables 1 and 2

Supplementary Figure 1

Supplementary Reference

Appendix of Million Women Study Collaborators

Benjamin J. Cairns,[1*] Ruth C. Travis,[1] Xiao-Si Wang,[1] Gillian K. Reeves,[1] Jane Green,[1] and Valerie Beral,[1] on behalf of the Million Women Study Collaborators[1,2]

[1] Cancer Epidemiology Unit, University of Oxford, Richard Doll Building, Roosevelt Drive, Oxford OX3 7LF

[2] Listed in Appendix

[*] Corresponding author. E-mail: ben.cairns@ceu.ox.ac.uk, Phone: +44 1865 289 673, Fax: +44 1865 289 610.

Supplementary Table 1. Age-adjusted* characteristics of study participants at baseline, according to frequency of napping during the day

	**Frequency of daytime napping**					
	**rarely/never**		**sometimes**		**usually**	
	(n = 439,780)		(n = 306,963)		(n = 48,495)	
**Characteristic**						
Age at baseline, yr, mean (SD)	59.2	(4.5)	60.2	(4.6)	60.9	(4.7)
Age at menarche, yr, mean (SD)	13.0	(1.6)	12.9	(1.6)	12.9	(1.6)
Age at first birth, yr, mean (SD)	24.1	(4.3)	24.0	(4.2)	23.8	(4.3)
Full-term pregnancies, mean (SD)	2.1	(1.2)	2.1	(1.2)	2.1	(1.3)
Sleep per day, hr, mean (SD)	7.20	(2.1)	7.41	(2.8)	8.00	(3.8)
Alcohol per day (g)	7.69	(14)	6.97	(14.3)	7.12	(15.4)
Height, cm, mean (SD)	162.3	(6.6)	162.3	(6.7)	162.1	(6.8)
Body mass index, kg/m^2, mean (SD)	25.7	(4.3)	26.7	(4.8)	27.3	(5.5)
Current smoker, %	11.1		12.5		15.3	
Strenuous physical activity, >0 hr/wk, %	61.2		58.8		52.3	
Menopausal hormone therapy, current, %	25.6		28.9		33.3	
Socioeconomic status, lowest quintile, %	15.1		18.2		21.9	
In full-time paid work, %	18.2		11.5		8.9	
**Follow-up for cancer incidence**						
Incident cancers	27,015		21,231		3,668	
Time at risk, yr (millions)	3.26		2.26		0.35	

* All values were adjusted for age at baseline except age at baseline, number of women, number of incident cancers, and time at risk.

Supplementary Table 2. Effects of adjustment by various factors on relative risks of all cancers according to frequency of daytime napping

	Total cancer risk according to frequency of daytime napping				
	rarely/never	sometimes		usually	
**Adjustment factor**	**(referent) RR**	**RR**	**(95% CI)**	**RR**	**(95% CI)**
**MINIMALLY ADJUSTED** *	1.00	1.10	(1.08, 1.12)	1.20	(1.16, 1.24)
**Additionally adjusted separately for:** †					
Sleep duration	1.00	1.09	(1.07, 1.11)	1.18	(1.14, 1.22)
Smoking status	1.00	1.08	(1.07, 1.10)	1.17	(1.13, 1.21)
Alcohol consumption	1.00	1.10	(1.08, 1.12)	1.19	(1.15, 1.24)
Body mass index	1.00	1.08	(1.06, 1.10)	1.17	(1.13, 1.22)
Strenuous physical activity	1.00	1.10	(1.08, 1.12)	1.19	(1.15, 1.24)
Use of menopausal hormone therapy	1.00	1.09	(1.07, 1.11)	1.19	(1.15, 1.23)
Socioeconomic status	1.00	1.09	(1.07, 1.11)	1.19	(1.15, 1.23)
**MULTIVARIABLE ADJUSTED** ‡	1.00	1.06	(1.04, 1.08)	1.11	(1.07, 1.15)
**Additionally adjusted separately for:** §					
Age at first birth	1.00	1.06	(1.04, 1.08)	1.11	(1.07, 1.15)
Parity	1.00	1.06	(1.04, 1.08)	1.11	(1.07, 1.15)
Paid work	1.00	1.06	(1.04, 1.08)	1.11	(1.08, 1.15)

* Stratified by age and region at recruitment.

† Analyses of relative risk according to napping frequency, stratified by age and region at recruitment, and further adjusted for each of the listed factors in turn: sleep duration (<6, 6, 7, 8, 9, 10+ hr per 24 hours); smoking status (never smoker, former smoker, or currently smoking 1-14, 15+ or unknown cigarettes/day); alcohol consumption (non-drinker, drinkers consuming <10, 10+ g/day); body mass index (<22.5, 22.5-24.9, 25-27.4, 27.5-29.9, 30+ kg/m^2); strenuous physical activity (none, 0.1-1, >1 hr per week); use of menopausal hormone therapy (never, past, current); and socioeconomic status (quintiles of Townsend deprivation index (Townsend *et al*, 1988) at recruitment). Categories were added to each covariate to classify women with missing data.

‡ Analysis adjusted for sleep duration, smoking, alcohol consumption, body mass index, strenuous physical activity, use of menopausal hormone therapy, and socioeconomic status.

§ As in the multivariable model, but additionally adjusted for each of the listed factors in turn: age at first birth (nulliparous, age <25 or 25+ years); parity (nulliparous, 1-2, 3+ full-term pregnancies); paid work (none, part-time, full-time). Categories were added to each covariate to classify women with missing data.

CANCER RISK ACCORDING TO FREQUENCY OF DAYTIME NAPPING

Cancer site or type (ICD−10 codes)	Number of cases	rarely/never (referent) RR	sometimes RR (95% CI)	usually RR (95% CI)	*P* (trend)
Mouth and pharynx (C00−C14)	570	1.00	1.08 (0.91, 1.29)	0.93 (0.65, 1.33)	0.77
Oesophagus (C15)	660	1.00	1.04 (0.89, 1.23)	0.93 (0.67, 1.28)	0.98
Stomach (C16)	574	1.00	1.13 (0.95, 1.34)	0.96 (0.68, 1.36)	0.49
Colon (C18)	3750	1.00	1.10 (1.03, 1.18)	1.07 (0.94, 1.23)	0.02
Rectal (C19−C20)	1827	1.00	1.04 (0.94, 1.14)	1.00 (0.82, 1.22)	0.63
Pancreas (C25)	1223	1.00	1.00 (0.89, 1.13)	1.07 (0.85, 1.34)	0.70
Lung (C34)	4143	1.00	1.10 (1.03, 1.17)	1.22 (1.09, 1.37)	<0.001
Melanoma (C43)	2140	1.00	0.94 (0.86, 1.03)	0.79 (0.64, 0.97)	0.02
Breast (C50)	20058	1.00	1.05 (1.02, 1.08)	1.11 (1.05, 1.18)	<0.001
Endometrium (C54)	3353	1.00	1.15 (1.07, 1.23)	1.27 (1.11, 1.46)	<0.001
Ovary (C56)	2504	1.00	1.08 (0.99, 1.17)	1.00 (0.84, 1.19)	0.22
Renal cell carcinoma (C64)	967	1.00	1.15 (1.00, 1.31)	1.22 (0.95, 1.57)	0.03
Bladder (C67)	738	1.00	0.99 (0.84, 1.15)	1.20 (0.91, 1.59)	0.45
Central nervous system (C70−72)	726	1.00	0.99 (0.85, 1.16)	1.00 (0.73, 1.36)	0.94
Non−Hodgkin's lymphoma (C82−C85)	2055	1.00	1.08 (0.98, 1.18)	1.22 (1.03, 1.45)	0.02
Multiple myeloma (C90)	747	1.00	1.12 (0.96, 1.30)	1.07 (0.79, 1.45)	0.23
Leukemia (C91−C95)	838	1.00	1.08 (0.94, 1.25)	1.38 (1.07, 1.78)	0.02
Other and unspecified	4862	1.00	1.05 (0.99, 1.12)	1.17 (1.04, 1.31)	0.005
**TOTAL CANCER (C00−C97)**	51914	1.00	1.06 (1.04, 1.08)	1.11 (1.07, 1.15)	<0.001

sometimes RR & 95% CI

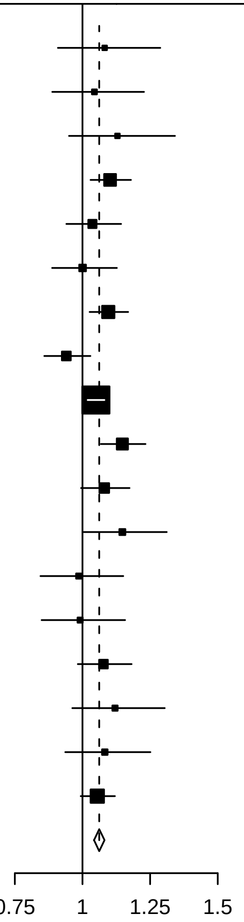

usually RR & 95% CI

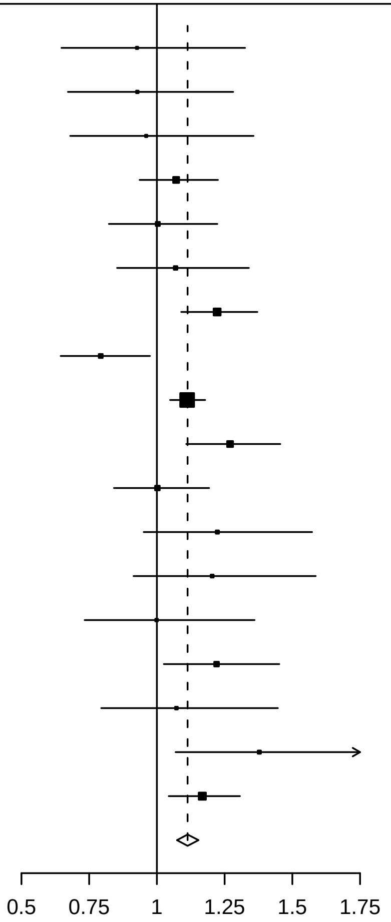

Supplementary Figure 1. Relative risks of cancers at specific sites, other invasive cancers and all invasive cancers combined, according to frequency of daytime napping.

Supplementary Reference

Townsend, P., P. Phillimore and A. Beattie (1988). Health and Deprivation: Inequality and the North. London, Croon Helm.

Appendix of Million Women Study Collaborators

Collaborating NHS Breast Screening Centres. Avon, Aylesbury, Barnsley, Basingstoke, Bedfordshire & Hertfordshire, Cambridge & Huntingdon, Chelmsford & Colchester, Chester, Cornwall, Crewe, Cumbria, Doncaster, Dorset, East Berkshire, East Cheshire, East Devon, East of Scotland, East Suffolk, East Sussex, Gateshead, Gloucestershire, Great Yarmouth, Hereford & Worcester, Kent (Canterbury, Rochester, Maidstone), Kings Lynn, Leicestershire, Liverpool, Manchester, Milton Keynes, Newcastle, North Birmingham, North East Scotland, North Lancashire, North Middlesex, North Nottingham, North of Scotland, North Tees, North Yorkshire, Nottingham, Oxford, Portsmouth, Rotherham, Sheffield, Shropshire, Somerset, South Birmingham, South East Scotland, South East Staffordshire, South Derbyshire, South Essex, South Lancashire, South West Scotland, Surrey, Warrington Halton St Helens & Knowsley, Warwickshire Solihull & Coventry, West Berkshire, West Devon, West London, West Suffolk, West Sussex, Wiltshire, Winchester, Wirral and Wycombe.

Million Women Study Steering Committee. Emily Banks, Valerie Beral, Ruth English, Jane Green, Julietta Patnick, Richard Peto, Gillian Reeves, Martin Vessey and Matthew Wallis.

Million Women Study Coordinating Centre. Simon Abbott, Naomi Allen, Miranda Armstrong, Angela Balkwill, Emily Banks, Vicky Benson, Valerie Beral, Judith Black, Anna Brown, Diana Bull, Benjamin Cairns, Kathy Callaghan, Karen Canfell, Dexter Canoy, James Chivenga, Barbara Crossley, Francesca Crowe, Dave Ewart, Sarah Ewart, Lee Fletcher, Toral Gathani, Laura Gerrard, Adrian Goodill, Jane Green, Lynden Guiver, Isobel Lingard, Elizabeth Hilton, Sau Wan Kan, Carol Keene, Oksana Kirichek, Mary Kroll, Nicky Langston,

Bette Liu, Maria-Jose Luque, Lynn Pank, Kirstin Pirie, Gillian Reeves, Andrew Roddam, Keith Shaw, Emma Sherman, Evie Sherry-Starmer, Helena Strange, Siân Sweetland, Alison Timadjer, Sarah Tipper, Ruth Travis, Xiaosi Wang, Joanna Watson, Lucy Wright, Owen Yang, Heather Young.
